# Structure–Function and Therapeutic Potential of Spider Venom-Derived Cysteine Knot Peptides Targeting Sodium Channels

**DOI:** 10.3389/fphar.2019.00366

**Published:** 2019-04-11

**Authors:** Fernanda C. Cardoso, Richard J. Lewis

**Affiliations:** Centre for Pain Research, Institute for Molecular Bioscience, The University of Queensland, St Lucia, QLD, Australia

**Keywords:** spider venoms, ICK peptides, voltage-gated ion channels, structure–activity relationship, novel drugs

## Abstract

Spider venom-derived cysteine knot peptides are a mega-diverse class of molecules that exhibit unique pharmacological properties to modulate key membrane protein targets. Voltage-gated sodium channels (Na_V_) are often targeted by these peptides to allosterically promote opening or closing of the channel by binding to structural domains outside the channel pore. These effects can result in modified pain responses, muscle paralysis, cardiac arrest, priapism, and numbness. Although such effects are often deleterious, subtype selective spider venom peptides are showing potential to treat a range of neurological disorders, including chronic pain and epilepsy. This review examines the structure–activity relationships of cysteine knot peptides from spider venoms that modulate Na_V_ and discusses their potential as leads to novel therapies for neurological disorders.

## Introduction

Animal venoms are an extraordinary source of bio-active peptides that modulate membrane proteins to facilitate prey capture and defense. Venomous spiders, cone snails, fish, sea anemones, wasps, scorpions, snakes and dinoflagellates produce small molecules and/or peptides exhibiting pharmacological properties of singular value for the research in pharmacological tools and novel drugs (Ziegman and Alewood, 2015; Dongol et al., 2016; Cardoso and Lewis, 2017; Kessler et al., 2017; Abraham and Lewis, 2018; Cardoso et al., 2018). These toxins modulate a range of receptors and channels, including VGIC, TRP, muscarinic and nicotinic acetylcholine receptors (mAChR and nAChR), ASIC, NET and G protein coupled receptors (GPCRs). Based on the number of species and venom complexity, spider venoms provide a mega-diverse source of bio-active cysteine knot peptides, many of which modulate Na_V_ with high potency and selectivity (Cardoso and Lewis, 2017).

**FIGURE 1:**
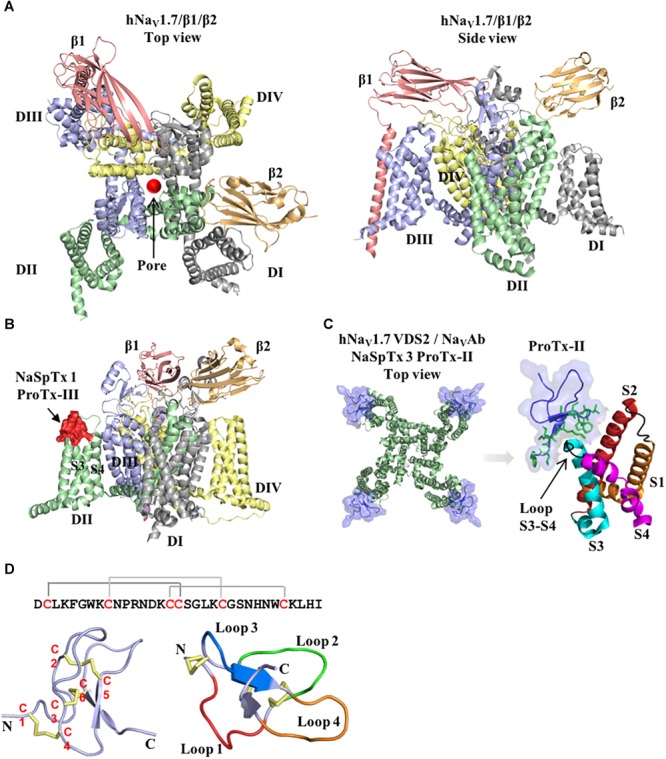
Structure of the voltage-gated sodium channel and the inhibitory cysteine knot (ICK) spider peptide. **(A)** Three-dimensional structure of the human Na_V_1.7 in the presence of the auxiliary subunits β1 and β2 determined by cryo-EM (PDB 6J8J) (Shen et al., 2019). Top and side views are presented. The domain I (DI) is colored in gray, domain II (DII) is colored in green, domain III (DIII) is colored in blue and domain IV (DIV) is colored in yellow. The auxiliary subunits β1 and β2 are colored in salmon and orange, respectively. **(B)** Representative binding site of a NaSpTx 1 in the DII S3-S4 loop of the hNa_V_1.7 channel (PDB 6J8J) (Shen et al., 2019). Domains and auxiliary subunits are colored as in **(A)**, and the NaSpTx1 peptide ProTx-III (PDB 2MXM) (Cardoso et al., 2015) is colored in red. **(C)** Detailed binding site of the NaSpTx 3 ProTx-II over the hNa_V_1.7 voltage–sensor domain 2 (VSD2)-Na_V_Ab chimeric channel (PBD 6N4I) (Xu et al., 2019). Top view of the hNa_V_1.7 VSD2-Na_V_Ab chimeric channel colored in green showing ProTx-II colored in blue bound to the voltage sensor domain 2 in close proximity with the S3–S4 loop. In the structure on the left, the segments S1 to S4 are colored in orange, red, cyan and magenta, respectively. The loops S1–S2 and S3–S4 are colored in black and the residues in the loop 4 and C-terminal of ProTx-II are represented by green sticks. **(D)** Typical structure of an inhibitory cysteine knot (ICK) peptide from spider. Primary and three-dimensional structure of ProTx-III (PDB 2MXM) (Cardoso et al., 2015) showing cysteine connectivity (C1 – C4, C2 – C5, and C3 – C6) and loops 1–4 colored in red, green, blue and orange, respectively.

Voltage-gated sodium channels (Na_V_1.1–1.9), in particular, are key players in the transmission of electrical signals in excitable cells and also involved in the pathophysiology of neurological disorders, including poorly treated conditions as chronic pain and epilepsy (Luiz and Wood, 2016; Klein-Weigel et al., 2018; Sloan et al., 2018; Szepetowski, 2018). The Na_V_ channels α-subunit comprises four domains (DI–DIV), each formed by six transmembrane segments (S1–S6), including S4 which contributes transmembrane voltage sensitivity, the tip of S5–S6 which contributes to sodium ion selectivity, and the intracellular loop connecting S6 of DIII and S1 of DIV which contributes to fast inactivation (Figure 1). The co-associated auxiliary β-subunits (β1–β4) are positioned above the VSD explaining their ability to influence channel gating (Figure 1A,B). Interestingly, human genetic studies disclosed mutations in Na_V_1.7 and Na_V_1.9 channels leading to congenital insensitivity to pain, a rare condition characterized by lack of physical pain (Cox et al., 2006; Phatarakijnirund et al., 2016), while gain-of-function mutations in Na_V_1.6, Na_V_1.7, Na_V_1.8, and Na_V_1.9 lead to painful neuropathies such as trigeminal neuralgia and erythromelalgia (Drenth and Waxman, 2007; Faber et al., 2012; Huang et al., 2014; Grasso et al., 2016). Genetic mutations in the Na_V_1.1 and Na_V_1.2 channels result in functional defects linked to epileptic syndromes (Meisler and Kearney, 2005; Thompson et al., 2011). Furthermore, altered Na_V_ channels function and expression are prominent in chronic inflammatory and neuropathic pains, with localization remodeling, altered expression and sensitization often observed in the subtypes Na_V_1.3, Na_V_1.6, Na_V_1.7, Na_V_1.8, and Na_V_1.9 (Cardoso and Lewis, 2017). Na_V_1.4 and Na_V_1.5 have restricted expression in the skeletal and cardiac muscle, respectively, which are important off-target pharmacologies to be considered when developing Na_V_ channel therapeutics.

The role of Na_V_ channels in both health and pathological pain have been further elucidated with the support of potent and selective Na_V_ channels modulators isolated from spider venoms. By using a subtype selective Na_V_1.1 activator isolated from the tarantula *Heteroscodra maculata*, the key role played by Na_V_1.1 in physiological mechanical pain (Osteen et al., 2016) and chronic visceral pain (Salvatierra et al., 2018) was established. More interestingly, pain relief is achieved in pre-clinical models of inflammatory and neuropathic pain administrated with Na_V_ channels inhibitors isolated from other spiders venoms, as for the ICK peptides ProTx-II (Tanaka et al., 2015; Flinspach et al., 2017), HnTX-IV (Liu et al., 2014a), Hl1a (Meng et al., 2016), HwTx-IV (Liu et al., 2014b), and Pn3a (Deuis et al., 2017).

The unique properties of spider ICK peptides in modulating ion channels give rise to opportunities for developing better and safer therapies targeting Na_V_ channels. This, in association to the current need for effective drugs to treat challenging neurological disorders and to overcome severe side-effects by opioid analgesic drugs foster the use of these bio-active spider peptides in therapeutics development. In this review, we examine recent advances in the SAR of cysteine knot peptides from spider venoms that inhibit Na_V_ and discuss their potential for the development of novel therapies.

## Structure–Activity Relationships of Spider ICK Peptides

Spiders are the largest group of venomous animals, with more the 40,000 species described to date (Platnick, 2014). Their venoms are rich in peptides that inhibit or activate Na_V_ channels by binding to domains outside the channel pore to allosterically promote opening or closing of the channel (Figure 1A–C) (Cardoso and Lewis, 2017). These binding sites include the VSD associated with domains II and IV that bind site 4 and 3 Na_V_ channel toxins, respectively. Interestingly, these peptides have a conserved ICK scaffold that confers high stability and resistance to high temperatures, low pH and digestion by proteases. A typical spider peptide ICK scaffold, with few exceptions, comprises three disulphide bridges C1–C4, C2–C5, and C3–C6 that fold these peptides into a globular structure with four distinct loops and an extended C-terminal tail (Figure 1D). These peptides were classified into distinct families of Na_V_ modulators named NaSpTx 1–12 based on their amino acids sequence and cysteine position (Klint et al., 2012), with extensive SAR studies reported for NaSpTx 1 and 3, and to a lesser extent for NaSpTx 7. Modern high throughput screening technologies using fluorescence-imaging and automated patch-clamp cell-based assays have facilitated the identification of peptides that display high potency and selectivity to modulate Na_V_ subtypes (Cardoso et al., 2015; Klint et al., 2015b). These peptides have been the focus of studies to unravel their pharmacological properties and potential for the development of novel and more effective drugs, studies which have been considerably advanced through investigations of the SAR of spider ICK peptides over Na_V_ channel subtypes. By using state-of-art methods for peptide production and detailed pharmacology characterization through patch-clamp electrophysiology in primary isolated neurons (e.g., DRG) or mammalian cells expressing Na_V_ channels subtypes, and X-ray and nuclear magnetic resonance (NMR) for determination of three-dimensional structure, these SAR studies have unraveled key features associated to modulation Na_V_ channels. Our current understanding of the SAR of each of the main ICK family peptides at Na_V_ channels are outlined below.

## NaSpTx 1

### GpTx-1

The ICK peptide GpTx-1 (μ/ω-TRTX-Gr2a) isolated from the tarantula *Grammostola rosea* was first described as a nanomolar inhibitor of Ca_V_ (Ono et al., 2011) and later as a nanomolar Na_V_ inhibitor from *Grammostola porter* (Murray et al., 2015). Its high potency for the sodium channel subtype Na_V_1.7 (IC_50_ of 10 nM) made it an attractive lead for SAR studies (Murray et al., 2015, 2016) (Table 1). Alanine scanning revealed the residues W29, K31 and F34 are essential for the Na_V_1.7 inhibition (Murray et al., 2015). In its native form, GpTx-1 is 20-fold and 1000-fold selective over Na_V_1.4 and Na_V_1.5, respectively, with the F5A mutant enhancing to 300-fold selectivity over Na_V_1.4. In the same study, additional positional substitutions using natural and non-natural amino acids other than alanine in the positions 5, 6, 26, and 28 put forward the rational design of an optimized GpTx-1 containing the substitutions F5A, M6F, T26L, K28R. This new GpTx-1 analog displayed 6-fold enhanced potency for Na_V_1.7 and 1000-fold selectivity over Na_V_1.4 and Na_V_1.5.

**Table 1:** Structure–activity relationship of spider ICK peptides and Na_V_ channels.

Peptide	Amino acids sequences	*In vitro* properties	References
***NaSpTx 1***			
GpTX-I	wt-**DCLGFMRKCIPDNDKCCRPNLVCSRTHKWCKYVF**	IC_50_ value of 4 nM for Na_V_1.7 and 68- and 950-fold selective over Na_V_1.4 and Na_V_1.5, respectively.	Murray et al., 2015
	**DCLGAMRKCIPDNDKCCRPNLVCSRTHKWCKYVF**	Improved 300-fold selectivity over Na_V_1.4.	
	**DCLGFFRKCIPDNDKCCRPNLVCSRLHRWCKYVF**	Improved 6-fold potency for Na_V_1.7 and 1000-fold selective over Na_V_1.4 and Na_V_1.5.	Murray et al., 2016
HwTx-IV	wt-**ECLEIFKACNPSNDQCCKSSKLVCSRKTRWCKYQI^∗^**	IC_50_ value of 26 nM for Na_V_1.7 and 6-, 13- and 15-fold selective over Na_V_1.2, Na_V_1.3, and Na_V_1.4, respectively.	Xiao et al., 2008b; Deng et al., 2013; Minassian et al., 2013
	**(Pyro)ECLEIFKACNPSNDQCCKSSKLVCSRKTRWCKYQI^∗^**	Confer irreversible inhibition for TTX-S Na_V_ currents in DRG.	Rong et al., 2013
	**ACLEIFKACNPSNDQCCKSSKLVCSRKTRWCKYQI**	Improved 2 to 4-fold potency for Na_V_1.7 and Na_V_1.2.	Minassian et al., 2013
	**ECLAIFKACNPSNDQCCKSSKLVCSRKTRWCKYQI**		
	**GCLGIFKACNPSNDQCCKSSKLVCSRKTRWCKWQI^∗^**	Improved potency at 42-fold for Na_V_1.7.	Revell et al., 2013
	**GCLGIFKACNPSNDQCCKSSKLVCSRKTRWCKWQI**	Improved potency at 15-fold for Na_V_1.7 and 4-fold for Na_V_1.2^#^.	Rahnama et al., 2017
	**ECLEIFKACNPSNDQCCKSSKLVCSRKTRWCKYQI^∗^**	Altered lipid binding in the cell membrane.	Henriques et al., 2016
CcoTx1	wt-**DCLGWFKSCDPKNDKCCKNYTCSRRDRWCKYDL**	IC_50_ value of 3 nM for Na_V_1.2, 75 nM for Nav1.7 and >300 nM for other Na_V_ subtypes (Na_V_1.6 was not tested).	Bosmans et al., 2006; Shcherbatko et al., 2016
	**DCLGMFKSCDPENDKCCKRLVCSRSHRWCKWKL**	IC_50_ value of 25 nM for Na_V_1.7 and selectivity of 6-fold over Na_V_1.2 and 4-fold over Na_V_1.6.	Shcherbatko et al., 2016
	**ICLGMFKSCDPENDKCCKRLVCSRSHRWCKWKL**	IC_50_ value of 11 (C-terminal carboxi) and 2 nM (C-terminal amide) for Na_V_1.7, and selectivity of 15-fold over Na_V_1.2 and 6- fold over Na_V_1.6.	
	**ICLGMFKSCDPENDKCCKRLVCSRSHRWCKWKL^∗^**		
	**(Pyro)ECLGIFKSCDPENDKCCYRLVCSKSHRWCKWKL^∗^**	IC_50_ value of 2.5 nM for Na_V_1.7, and selectivity of 80-fold over Na_V_1.2 and 20-fold over Nav1.6, and extra 1.5-fold of irreversible binding for Na_V_1.7.	
HNTX-I	wt-**ECKGFGKSCVPGKNECCSGYACNSRDKWCKVLL**	No activity over Na_V_ channels	Klint et al., 2015a; Zhang et al., 2018b
	**ECKGFWKSCVPGKNECCSGYACSSRDKWCKVLL**	IC_50_ value of 440 nM for Na_V_1.7	Klint et al., 2015a
	**GCKGFGKSCVPGKNECCSGYACSSRHKWCKVWL**	IC_50_ value of 36 nM for Na_V_1.7	Zhang et al., 2018b
HNTX-III	wt-**GCKGFGDSCTPGKNECCPNYACSSKHKWCKVYL**	IC_50_ value of 150 nM for Na_V_1.7	Zhang et al., 2015
HNTX-IV	wt-**ECLGFGKGCNPSNDQCCKSSNLVCSRKHRWCKYEI^∗^**	IC_50_ value of 34 nM for Na_V_1.7	Li et al., 2004
***NaSpTx 3***			
ProTx-II	wt-**YCQKWMWTCDSERKCCEGMVCRLWCKKKLW**	IC_50_ value of 0.3–1 nM for Na_V_1.7 and 26-146 nM for the other Na_V_ subtypes (Na_V_1.1 was not tested).	Priest et al., 2007; Smith et al., 2007; Schmalhofer et al., 2008; Park et al., 2014
	**YCQKWMWTCDSERKCCEGMVCRLWCKKKLW-NHCH_3_**	IC_50_ value of 42 pMol for Na_V_1.7, selectivity of 83-fold over Na_V_1.2.	Park et al., 2014
	**GPYCQKWMQTCDSERKCCEGMVCRLWCKKKLL**	Improved selectivity over Na_V_1.4 and Na_V_1.5.	Flinspach et al., 2017
	**YCQKWMWTCDSERKCCEGMVCRLWCKKKLW**	Altered lipid binding in the cell membrane.	Agwa et al., 2017
JZTX-V	wt-**YCQKWMWTCDSKRACCEGLRCKLWCRKII^∗^**	IC_50_ value of 0.6 nM for Na_V_1.7, and selectivity of 4- and 4000-fold over Na_V_1.4 and Na_V_1.5, respectively.	Moyer et al., 2018
	**YCQKWMWTCDSKRACCEGLRCKLWCRKEI^∗^**	Improved selectivity over Na_V_1.4 to 500-fold (AM-8145).	
	**(Pra)YCQKWMWTCDSKRACCEGLRCKLWCRKEI^∗^**	Improved selectivity to 300- and 6000-fold over Na_V_1.4 and Na_V_1.5, respectively.	
	**(CyA)YCQKWMWTCDSKRACCEGLRCKLWCRKEI^∗^ (Pra)**	Improved selectivity to 128- and 1200-fold over Na_V_1.4 and Na_V_1.5, respectively.	
***NaSpTx 7***			
JZTX-III	wt-**DGECGGFWWKCGRGKPPCCKGYACSKTWGWCAVEAP**	IC_50_ value of 348 nM for Na_V_1.5, not active over other Na_V_ subtypes	Rong et al., 2011
	**DGECGGFWWKCGEGKPPCCKGYACSKTWGWCAVEAP**	Improved potency by 11-fold for Na_V_1.5.	

*Key amino acids residues identified in SAR studies of GpTx-1, HwTx-IV, CcoTx1, ProTx-II, JZTX-V, and JZTX-III which are involved in the potency and/or selectivity of ICK peptides for the Na_V_ channels family are described, along with the NaSpTx family of these peptides. Cysteines are colored in gray. Amino acids residues colored in red play a key role in potency over Na_*V*_ channels. Amino acids substitutions colored in blue lead to improvement in potency over key Na_*V*_ channels subtypes, and colored in green lead to improvement in selectivity over key Na_*V*_ off-targets. Amino acids residues colored in orange play a key role in lipid cell membrane binding. Asterisk (^∗^) denotes C-terminal amidation. ^#^Compared to Minassian et al. (2013). wt, wild-type.*

Later, another positional scan using glutamic acid, arginine, lysin, tryptophan and 1-naphthylalanine substitutions supported previous alanine scanning observations and revealed a number of new positions in GpTx-1 essential for the Na_V_1.7 activity, all together these key residues are F5, M6, S24, H27, W29, K31, Y32, and F34 (Murray et al., 2016). Contrary to alanine substitutions, most of the substitutions with 1-naphthylalanine didn’t produce folded peptides, with few similar cases for substitutions with tryptophan, followed by lysine, arginine and glutamic acid. Furthermore, the importance of S24 was observed only in the S24E mutant, being all other substitutions at position 24 unable to produce folded peptides. In the three-dimensional structure of GpTx-1, these identified active residues form a cluster on the surface allowing a direct interaction with Na_V_1.7 (Murray et al., 2016). These potential interactions were further investigated using a homology model of the hNa_V_1.7 channel docked with the NMR structure of GpTx-1, which predicted interactions of the residues F5 and K31 of GpTx-1 with I767 and E811 located in the domain II voltage sensor region, respectively. Other potential electrostatic interactions were predicted between R25 and R7 of GpTx-1 and E759 and E818 of the ion channel, respectively. Overall, the optimization of GpTx-1 toward an increase in potency for Na_V_1.7 and selectivity over Na_V_1.4 and Na_V_1.5 was possible with the addition of a hydrophobic aromatic residue at position 6 (substitution M6F), and a hydrophobic residue at position 26 (substitution T26L). A summary of the SAR for GpTx-1 is shown in Table 1.

### HwTx-IV

**FIGURE 2:**
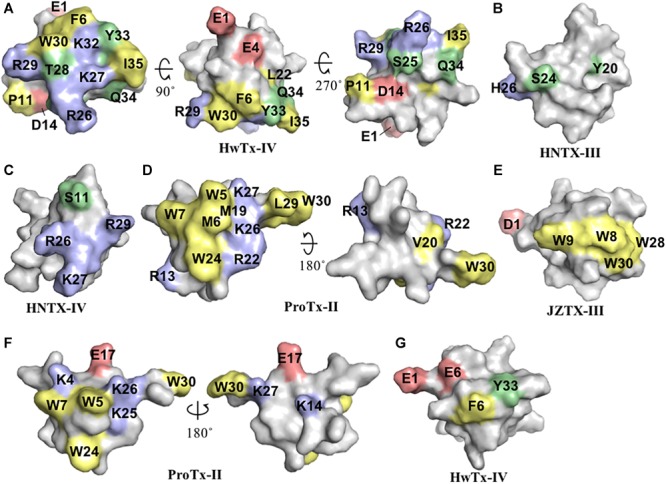
Three-dimensional structure of spider ICK peptides displaying key residues involved in the inhibition of Na_V_ channels and cell membrane binding. **(A)** Three-dimensional structure of HwTx-IV determined by NMR (PBD 2m4x) (Minassian et al., 2013), **(B)** HNTX-III determined by NMR (PBD 2jtb), **(C)** HNTX-IV determined by NMR (PBD 1niy) (Li et al., 2004), **(D)** ProTx-II determined by X-Ray (PBD 5o0u) (Wright et al., 2017), and **(E)** JZTX-III determined by NMR (PBD 2i1t). The labeled amino acids residues have key role in potency over Na_V_ channels and lead to loss in activity as described in the text and Table 1. **(F)** Structure of ProTx-II determined by X-Ray (PDB 5o0u) (Wright et al., 2017) and **(G)** Structure of HwTx-IV (PDB 2m4x) (Minassian et al., 2013) determined by NMR. The labeled amino acids residues have key role in cell membrane binding as described in the text and Table 1. Amino acids residues are colored as follow: yellow for hydrophobic, red for acid, blue for basic and green for neutral. All three-dimensional structures were prepared in PyMOL (DeLano, 2002).

Huwentoxin-IV (μ-TRTX-Hs2a), isolated from the venom of the Chinese bird spider *Selenocosmia huwena*, was first identified as a potent (IC_50_ of 30 nM) Na_V_ inhibitor of TTX-S currents in DRG neurons (Peng et al., 2002). This inhibitor has been the subject of extensive SAR studies (Table 1 and Figure 2). Initial SAR observations for HwTx-IV described a naturally occurring toxin variant with an N-terminal pyroglutamate instead of glutamate that had unchanged potency despite its irreversibility at strong depolarizing potentials (e.g., +200 mV) in DRG neurons and in Na_V_1.7 expressed in HEK293 cells (Rong et al., 2013). More detailed SAR studies of HwTx-IV revealed that C-terminal mutations T28D, R29A or Q34D reduced Na_V_ potency in DRG neurons (Deng et al., 2013), while an alanine scan of HwTX-IV additionally revealed W30 and K32 also critical for high affinity interactions at Na_V_1.2 and Na_V_1.7 (Minassian et al., 2013) (Figure 2A). In this latter study, molecular dynamics simulations revealed substitutions displaying loss of potency for Na_V_1.2 and Na_V_1.7 also led to an increase in the flexibility of the loops 2 (substitutions P11A and D14A), in loop 3 (substitutions F6A, L22A, W30A, and Y33A) and in loop 4 (substitutions R26A and K27A), while the substitutions S25A, K32A, and I35A reduced HwTx-IV potency for Na_V_1.2 and Na_V_1.7 without affecting its structure and loops flexibility (Minassian et al., 2013).

**FIGURE 3:**
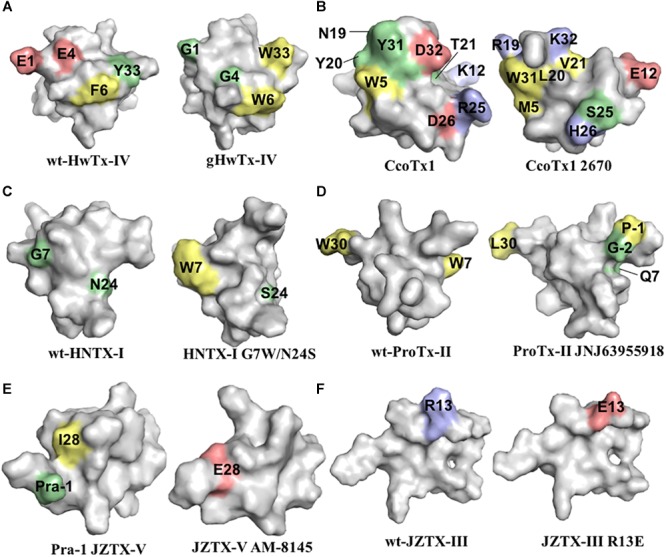
Three-dimensional structure of spider ICK peptides displaying key residues involved in the enhancement of activity for Na_V_ channels, or enhancement of selectivity for Na_V_ channels off-targets. **(A)** Structures of HwTx-IV (PDB 2m4x) (Minassian et al., 2013) and gHwTx-IV (PDB 5tlr) (Agwa et al., 2017) determined by NMR. **(B)** Structures of CcoTx1 determined by NMR (PDB 6br0) (Agwa et al., 2018) and the derived analog 2670 determined by X-ray (PDB 5epm) (Shcherbatko et al., 2016). **(C)** Structures of HNTX-I (PDB 2mqf) and the derived analog G7W/N24S (PDB 2mxo) determined by NMR (Klint et al., 2015a). **(D)** Structures of ProTx-II determined by X-Ray (PDB 5o0u) (Wright et al., 2017) and derived analog JNJ63955918 determined by NMR (PDB 5tcz) (Flinspach et al., 2017). **(E)** Structure of analogs Pra-1 JZTX-V (PDB 6chc) and AM-8145 (6cgw) determined by NMR (Moyer et al., 2018). **(F)** Structure of JZTX-III determined by NMR (PDB 2i1t) (Liao et al., 2006) and modeling of the derived analog R13A using SWISS-MODEL (Arnold et al., 2006). Amino acids residues are colored as follow: yellow for hydrophobic, red for acid, blue for basic and green for neutral. Amino acids substitutions or additions labeled in the respective analogs are involved in the improvement of Na_V_ inhibitory activity or selectivity as described in the text and Table 1. All three-dimensional structures alignments were prepared in PyMOL (DeLano, 2002).

Further alanine scanning of HwTx-IV identified E1, E4, F6, and Y33 as important contributors for Na_V_1.7 affinity (Revell et al., 2013). These findings afforded the rational design and optimization of HwTx-IV with increased potency for Na_V_1.7 and maintaining low affinity for the off-target Na_V_1.5 (Figure 3). Among these new HwTx-IV analogs, the mutant HwTx-IV-G1/G4/W33 demonstrated the highest increase in activity, with 42-fold enhanced potency for Na_V_1.7 inhibition, followed by HwTx-G1/G4 and HwTx-IV-A1/A4/W33 (Revell et al., 2013). More recently, the activity of HwTx-IV-G1/G4/W33 was tested over other members of the Na_V_ family, showing an increase in inhibition of 12-fold for Na_V_1.2 and 47-fold for Na_V_1.3 compared to wild-type HwTx-IV, but maintained the low potency for the off-target Na_V_1.4 and Na_V_1.5 (Xiao et al., 2008a; Rahnama et al., 2017). Altogether, the residues F6, P11, D14, L22, T28, R29, W30, K32, Y33, and Q34, when mutated, led to a loss of affinity of HwTx-IV for Na_V_ channels (Table 1 and Figure 3A). Furthermore, the optimization of HwTx-IV activity for Na_V_1.7 was possible with the removal of acid negative residues at the N-terminal (substitutions E1 to A or G), and increase in hydrophobicity at the C-terminal (substitution Y33W) (Table 1 and Figure 3A). These produced a new surface in the HwTx-IV three-dimensional structure with increased polarity and hydrophobicity.

### CcoTx1

CcoTx1 (β-TRTX-Cm1a) is a potent Na_V_ inhibitor isolated from the tarantula *Ceratogyrus cornuatus* (Bosmans et al., 2006). It has strong preference for the subtype Na_V_1.2 and lower affinity for the off-targets Na_V_1.4 and Na_V_1.5 (Table 1). The SAR of CcoTx1 was investigated using a combination of direct evolution, saturation mutagenesis, chemical modifications and rational drug design to unravel key residues involved in potency and selectivity of this peptide over the Na_V_ family (Shcherbatko et al., 2016). Using direct evolution, CcoTx1 was optimized to improve its potency for the subtype Na_V_1.7, and off-target selectivity for Na_V_1.2 and Na_V_1.6, producing the variant named 2670. This mutant contained the substitutions W5M, K12E, N19R, Y20L, T21V, R25S, D26H, Y31W, and D32K (Table 1 and Figure 3B).

Further SAR investigations aiming to improve selectivity and potency for Na_V_1.7 were performed using saturation mutagenesis, revealing that substitutions at the N- and C-terminal regions, along with positions 20 and 21 of 2670, were not well tolerated. Combining this SAR information, the Na_V_1.7 pharmacophore of 2670 was defined by the hydrophobic residues M5, F6, W28, W31, and L33, and polar or positively charged residues R19, H26, K30, and K32. Saturation mutagenesis substitutions that conferred improved selectivity over off-targets and didn’t alter the Na_V_1.7 inhibitory potency were K18Y, R24K, and R27N. Still using this approach, a new mutant was identified with one single substitution D1I that conferred improved activity and selectivity (Table 1). C-terminal amidation of 2670 (2670a) and D1I (D1Ia) improved potency for Na_V_1.7 which was accompanied by an increase in potency for Na_V_1.6, but not for Na_V_1.2. In addition, a D1Ia variant containing a terminal pyroglutamate (D1Za) improved binding to Na_V_1.7 by 27% at strong depolarizing potentials, and maintained the same potency as D1Ia. This observation resembles the SAR properties of HwTx-IV (Rong et al., 2013). Overall, the optimization of CcoTx-1 activity toward Na_V_1.7 was possible with the replacement of acid negative residues at both N- and C-terminal to residues with increased hydrophobicity and positive charges, and with modifications toward the C-terminal that included substitutions for aromatic residues H and W. This led to a considerable change in the CcoTx-1 surface to include more hydrophobic and positively charged residues (Figure 3B).

### Hainantoxin

Hainantoxins (HNTXs) are ICK peptides comprised in the venom of the Chinese bird spider *Selenocosmia hainana*. Among these, the HNTX-I (μ-TRTX-Hhn2b) has weak to no activity over Na_V_ channels. SAR studies to restore the Na_V_ inhibitory activity of HNTX-I were performed using rational design based on other members of the NaSpTx 1 (Klint et al., 2015a; Zhang et al., 2018b) (Table 1 and Figure 3C). The substitutions G6W/N23S/W28F and G6W/N23S produced analogs with IC_50_ values of 1 and 0.44 μM for Na_V_1.7, respectively (Klint et al., 2015a). Later, the analogs N23S/D26H, N23S/D26H/L32W and E1G/N23S/D26H/L32W showed IC_50_ values of 79, 71, and 36 nM for Na_V_1.7, respectively. In this study, the motif X_1_X_2_SWCKX_3_ was identified as critical for the inhibitory activity for Na_V_1.7. Altogether, the HNTX-I activity for Na_V_1.7 was restored by removal of acid negative residues at both N- and C-terminal, and the addition of residues with increased hydrophobicity and positive charges, including the aromatic residues H and W.

Another hainantoxin, HNTX-III (μ-TRTX-Hhn2a), is an inhibitor of Na_V_1.7 with IC_50_ value of 232 nM (Liu et al., 2013). The SAR of this peptide was performed consequent to the discovery of several isoforms variants present in the transcriptome of the venom gland of this spider (Zhang et al., 2015) (Table 1). The pharmacological properties of these variants were tested, revealing that the substitutions Y20H, S24N, H26D and Y20H/S24N were all detrimental to the Na_V_1.7 activity (Figure 2B). The last hainantoxin discussed is HNTX-IV (μ-TRTX-Hhn1b), a potent inhibitor of TTX-S currents in DRG neurons (IC_50_ of 34 nM; Li et al., 2004). In this study, the SAR of HNTX-IV revealed K27 and R29 residues positions involved in the Na_V_ activity (Table 1 and Figure 2C). More specifically, the mutants S12A and R26A produced IC_50_ values of 58 and 96 nM, respectively, while K27A and R29A produced IC_50_ values of 3.2 and 7 μM, respectively. This work reinforces the essential role of positively charged residues located in the surface of spider ICK peptides for Na_V_1.7 inhibition.

## NaSpTx 3

### ProTx-II

ProTx-II (β/ω-TRTX-Tp2a) is amongst the most potent Na_V_ inhibitors described to date, with reported IC_50_ value of 0.3 nM for inhibition of Na_V_1.7 (Schmalhofer et al., 2008). This toxin was isolated from the spider *Thrixopelma pruriens* using rNav1.8 assay guided fraction that evaluated the inhibitory properties for the Na_V_1.8 channel of individual venom fractions separated by cation exchange liquid chromatography (Middleton et al., 2002). Despite of its exquisite inhibitory potency for Na_V_1.7, it also potently inhibits other members of the Na_V_ family, including the off-targets Na_V_1.4 and Na_V_1.5. The SAR of ProTx-II was initially investigated over the Na_V_1.5 channel (Table 1 and Figure 2D). Analogs of ProTx-II produced by recombinant expression and chemical synthesis containing alanine or glutamine substitutions revealed a peptide active face composed of hydrophobic and cationic residues (Smith et al., 2007). More specifically, ten of these analogs showed losses in potency from 10- to 125-fold, with major losses associated with W5A and K26A substitutions. The positively charged residues substitutions K27Q, R13Q, and R22A led a significant loss in potency, while neutralization of negatively charged residues didn’t affect the Na_V_1.5 inhibition. Furthermore, the substitutions M6A, W7A, M19L, V20A, W24L, L29A, and W30A each produced a > 10-fold loss in potency. Similarly, another SAR study of ProTx-II identified the residues in the hydrophobic face essential for Na_V_1.5 activity, including W5, M6, W7, W24, while residues identified as not critical for inhibitory activity included Y1, Q3, T8, N10, S11, E12, E17, and L23 (Priest et al., 2007).

SAR of ProTx-II has also been investigated over the channels Na_V_1.2 and Na_V_1.7 (Park et al., 2014) (Table 1 and Figure 2D). In this study, besides the identification of residues mutations detrimental to Na_V_ activity such as substitution of C-terminal KLW to II, an optimized analog ProTx-II-NHCH_3_ with ∼23-fold greater potency for Na_V_1.7 was identified. In this same work, the ICK peptide Phrixotoxin I, also known as PaTx I, isolated from the tarantula *Phrixotrichus auratus*, and a potent K_V_ inhibitor with IC_50_ value of 28 nM was submitted to a SAR study over Na_V_ channels (Diochot et al., 1999). It differs from ProTx-II by only 4 amino acids residues, and has moderate potency for Na_V_ channels, with IC_50_ value of 423 nM for Na_V_1.7, and no activity for Na_V_1.2 (Park et al., 2014). These naturally occurring differences were useful for the understand of the ProTx-II SAR over Na_V_ channels. The PaTx I C-terminal substitutions I28K and I29K, and addition of L30 and W31 (actual ProTx-II C-terminal) lead to an increase of 85-fold in the Na_V_1.7 potency, and an IC_50_ value of 45 nM for Na_V_1.2. This work unraveled a key role of the C-terminal for activity of NaSpTx 3 peptides, and describes interesting approaches for the improvement of Na_V_ activity through C-terminal modifications.

More recently, a detailed SAR of ProTx-II over Nav channels was investigated by 1500 toxin-derived peptides to identify a double mutant with extended N-terminal that maintained the inhibitory properties for Na_V_1.7 but lost affinity for Na_V_1.4 and Na_V_1.5 (Flinspach et al., 2017) (Table 1 and Figure 3D). This mutant, named JNJ63955918, contained additional G-2 and P-1 at the N-terminal and the substitutions W7Q and W30L. The interactions of ProTx-II with lipid membranes as well as with the hNav1.7 channels were investigated, revealing ProTx-II does interact with the cell membrane as part of its strategy to inhibit the Na_V_1.7 channel (Henriques et al., 2016) (Figure 3). In this study, substitutions of residues K to R and W to Y lead to a reduction in the potency of ProTx-II for hNa_V_1.7. The ProTx-II lipid interactions will be discussed in more details later in this review. Altogether, the residues W5, M6, W7, R13, M19, V20, R22, W24, K26, K27, L29, and W30, when mutated, led to a loss of affinity of ProTx-II for Na_V_ channels (Table 1 and Figure 2D). Interestingly, the optimization of ProTx-II toward a potent and selective Na_V_1.7 inhibitor was possible through the extension of the N-terminal with neutral and positively charged residues, and introduction of a positively charged group in the C-terminal.

### JZTX-V

Isolated from the tarantula *Chilobrachys jingzhao*, JZTX-V (β-TRTX-Cg2a) is a potent Na_V_1.7 inhibitor with IC_50_ value of 0.6 nM (Moyer et al., 2018). The SAR of JZTX-V over the subtypes Na_V_1.4, Na_V_1.5, and Na_V_1.7 was investigated using alanine or glutamic acid positional scanning to unravel key residues involved in Na_V_ inhibition, and to produce new mutants with improved potency and selectivity (Table 1 and Figure 3E). Substitutions that contribute to loss of the Na_V_1.7 inhibitory activity observed by alanine substitutions were W5, L19, W24, and R26, and by glutamic acid substitutions were M6, T8, D10, R13, and L23. Interestingly, a key substitution that improved off-target selectivity of JZTX-V over Na_V_1.4 was I28E. This mutation was able to induce a change in the conformation of JZTX-V to produce 500-fold selectivity and maintain the potency for Na_V_1.7.

In the same study, further investigation of the SAR of JZTX-V was performed through the introduction of side-chains containing residues such as Pra and β-cyanoalanine (CyA). Positions showing minimum involvement in Na_V_1.7 inhibitory activity disclosed by the glutamic acid scanning were Y1, S11, A14, and E17, and therefore selected to test this approach. The addition of Pra at the N-terminus of I28E created a new analog named AM-8145 with improved selectivity for Na_V_1.4 (300-fold) and Na_V_1.5 (6000-fold) and maintained potency for Na_V_1.7. Similar results were found for the addition of CyA at the N-terminus of I28E (analog AM-0422) and substitutions in the other selected positions. These properties were not present in the wild-type JZTX-V containing the Pra additions, but only in the I28E mutant. In summary, the optimization of JZTX-V toward a more selective Na_V_1.7 inhibitor was possible through the extension of the N-terminal with neutral or hydrophobic residues containing side-chains and addition of negative charge at the C-terminal. Interestingly, JZTX-V share similar key residues with ProTx-II essential for the inhibition of Na_V_1.7, as for W5, M6, R26, and W24 in JZTX-V, and selectivity optimization for Na_V_1.4 and Na_V_1.5 through N-terminal extensions.

## NaSpTx 7

### JZTX-III

The spider peptide JZTX-III (β/κ-TRTX-Cg1a), also isolated from the tarantula *Chilobrachys jingzhao*, was first characterized as a potent inhibitor of TTX-R currents in rat cardiac myocytes with IC_50_ value of 380 nM (Xiao et al., 2004), and later found to also inhibit K_V_2.1 channels (Yuan et al., 2007). The SAR of JZTX-III with Na_V_1.5 channels using alanine substitutions revealed residues D1, E3 and W8, W9, W28, and W30 as key players for its Na_V_1.5 inhibition (Rong et al., 2011) (Table 1 and Figure 2E). In addition, the substitution R13E enhanced the Na_V_1.5 inhibition by 11-fold (Figure 3F). Interestingly, JZTX-III does not inhibit the Na_V_1.7 channel, and this lack of affinity was associated to the residue D816 in the Na_V_1.7. The substitution D816R (corresponding to the R800 in the Na_V_1.5 channel) enhanced significantly the inhibitory properties of JZTX-III for Na_V_1.7.

## Interactions With the Cell Membrane

Spider ICK peptides are known to interact with the lipids in the cell membrane. Early studies showed these peptides are water-soluble and bind to the aqueous-exposed extracellular surface of ion channels, and surprisingly reach the target by partitioning into the lipid membrane (Lee and MacKinnon, 2004). This strategy allows the peptide to reach the voltage-sensor and enhance high-affinity inhibition. The ability to bind to lipids seems exclusive of gating modifiers ICK peptides binding to site 4 of Na_V_ channels (Smith et al., 2005). Furthermore, the Na_V_ VSD s are affected by the cell membrane lipid composition, with changes from native lipids to sphingomyelin altering G-V relations and affinity of ProTx-I for the domain II and domain IV S3-S4 loops (Milescu et al., 2009).

Detailed studies of these interactions using NMR revealed that ICK peptides can interact with the headgroup region of lipid membrane to induce a thinning of the bilayer (Mihailescu et al., 2014). In this interaction, many basic residues are positioned toward the aqueous phase, the W residues adopt an interfacial position and hydrophobic residues are in direct contact with the membrane. The SAR of ProTx-II, membrane lipids and Na_V_1.7 confirmed the previous observations, and revealed the ProTx-II analog E17K had increased on-rate for Na_V_1.7 compared to wild-type, but had no changes in the Na_V_1.7 potency (Henriques et al., 2016) (Table 1 and Figure 2F). Interestingly, all mutations at residues W to Y and K to R lead to a lower affinity for cell membranes. Changes in the structure of ICK peptides can also occur in the presence of lipids. This was observed in structural studies of ω-Aga-IVA (a Ca_V_ channel inhibitor ICK peptide) that undergoes changes in the presence of micelles (Ryu et al., 2017). More specifically, the C-terminal tail of ω-Aga-IVA assume a β-turn like conformation which is disordered in water.

The HwTx-IV mutant E1G/E4G/F6W/Y30W had increased affinity for the lipid membrane and higher potency for the inhibition of hNa_V_1.7 compared to wild-type (Agwa et al., 2017) (Table 1 and Figure 2G). Interestingly, the introduction of a PyroE in the N-terminal for HwTx-IV didn’t alter the membrane interactions and potency for Na_V_1.7. This confirmed previous observations for the naturally occurring PyroE-HwTx-IV that binds irreversibly to the Na_V_ channel voltage-sensor but at same potency of wild-type HwTx-IV (Rong et al., 2013). Although membrane interactions are key for the high affinity binding of ICK peptides over ion channels, such interactions have to-date had little influence on the selectivity for the Na_V_ channel family (Agwa et al., 2018).

## Binding Sites on Na_V_ Channels

The binding sites of spider ICK peptides at mammalian Na_V_ channels are starting to be characterized (Cardoso and Lewis, 2017). These gating-modifier toxins are known for their ability to change the voltage-dependence of activation and inactivation of Na_V_ channels to either inhibit or activate Na^+^ currents. Overall, they have preference for binding to the site 4 in domain II of the Na_V_ channel to trap the voltage–sensor and inhibit Na^2+^ currents, and to site 3 in domain IV to slow channel inactivation and maintain Na^2+^ currents. These sites have been reported for a range of spider ICK peptides, including HwTx-IV, ProTx-I, ProTx-II, PaurTx3, CcoTx-1, Hd1a and Df1a that inhibit Na_V_ channels (Bosmans et al., 2008; Xiao et al., 2008a; Klint et al., 2015b; Shcherbatko et al., 2016; Cardoso et al., 2017) and to SGTx1 and Hm1a that slow Na_V_ channels inactivation (Bosmans et al., 2008; Osteen et al., 2016).

The crystal structure of the related double-ICK spider peptide Dc1a bound to the Na_V_PaS was determined (Shen et al., 2018). Dc1a binds to a cleft between the VSD II and the pore loop of domain III of the Na_V_PaS and induce minimal changes in the channel structure. On the other hand, the Dc1a peptide undergoes considerable rearrangement to achieve its fit into the cleft. This information brings new insights into the docking and binding of other ICK peptides into the mammalian channel, which although widely reported to bind to domain II and domain IV S3-S4 loops of mammalian Na_V_ channels, could also be interacting with other proximal regions of the channel to reaches its final fit and maximal activities.

More recently, the cryo-EM structure of the human Na_V_1.7 channel was described in association of the auxiliary β1 and β2 subunits (Figure 1A,B) and the spider peptides HwTx-IV and ProTx-II (Shen et al., 2019). This study confirmed previous observations that HwTx-IV binds to the voltage-sensor of DII site 4 (Xiao et al., 2008a, 2010) and that ProTx-II binds to the voltage-sensors of DII and DIII sites 4 and 3 (Bosmans et al., 2008; Xiao et al., 2010). The cryo-EM structure of a chimeric hNa_V_1.7-Na_V_Ab channel bound to ProTx-II was also described recently (Figure 1C) (Xu et al., 2019), which confirmed ProTx-II binds to the voltage-sensor of DII site 4 of the Na_V_1.7 channel (Xiao et al., 2010). Although the determination of the three-dimensional structure of spider ICK peptides using X-ray or NMR are essential for the understanding of the SAR of these peptides, additional conformational changes are likely to happen for most of these ICK peptides when they achieved their final bound conformation with the Na_V_ channel and associated membrane lipids.

## Sar Integration of Spider ICK Peptides

**FIGURE 4:**
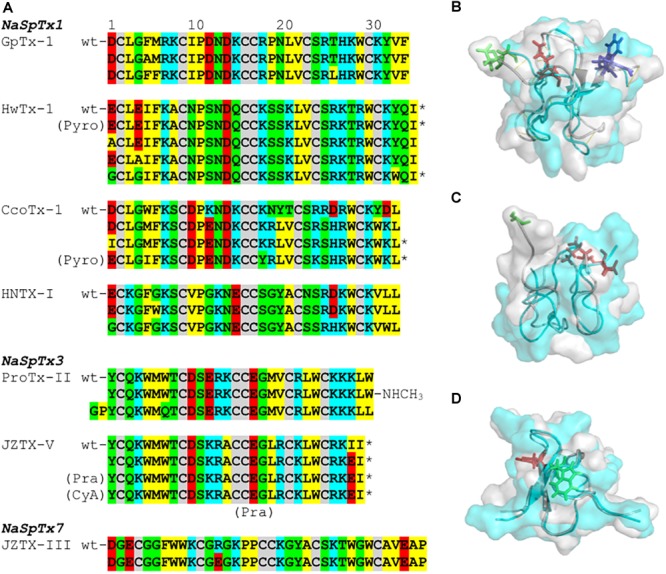
SAR integration of spider ICK peptides. Primary and three-dimensional structures comparison of spider ICK peptides belonging to NaSpTx families 1, 3, and 7. **(A)** Primary structure alignment of wild-type peptides and respective optimized analogs showing enhancement of activity and/or selectivity of Na_V_ channels. The amino acids residues are highlighted as follow: yellow for hydrophobic, red for acid, blue for basic, green for neutral, and gray for the cysteines. **(B)** Alignment of the three-dimensional structures of HwTx-IV (PDB 2m4x) (Minassian et al., 2013) colored in gray and gHwTx-IV (PDB 5tlr) (Agwa et al., 2017) colored in cyan. Structures are represented by cartoon and surface, and substitutions E1G, E4G and Y32W are represented by sticks colored in green, red, and blue, respectively. **(C)** Alignment of the three-dimensional structures of Pra-1 JZTX-V (PDB 6chc) colored in gray and AM-8145 (6cgw) colored in cyan (Moyer et al., 2018). Structures are represented by cartoon and surface, and the Pra-1 addition and substitution I28E are represented by sticks colored in green and red, respectively. **(D)** Alignment of the three-dimensional structures of HNTX-I (PDB 2mqf) colored in gray and the derived analog G7W/N24S (PDB 2mxo) colored in cyan (Klint et al., 2015a). Structures are represented by cartoon and surface, and substitutions G7W and N24S are represented by sticks colored in green and red, respectively. All three-dimensional structures alignments were performed in PyMOL (DeLano, 2002). Asterisk (^∗^) denotes C-terminal amidation.

Prevalent features in the SAR of spider ICK peptides and Na_V_ channels have been disclosed (Figure 4A and Table 1). Amongst the remarkable positions associated to Na_V_ activity in the NaSpTx 1 toxins are the residues 5 and 6, which are typically occupied by the hydrophobic residue phenylalanine. Similarly, NaSpTx 3 toxins typically have positions 5 and 6 occupied by hydrophobic residues W and M, which are also critical for Na_V_ inhibitory activity. Another interesting aspect of these SAR studies is apparent in optimized analogs with increased Na_V_ potency and selectivity that often display a decrease in negative charge at the N-terminal compared to wild-type toxins. This is supported by the introduction of Pyro, Pra, CyA and the residues G and P at the N-terminal in optimized peptides belonging to NaSpTx families 1 and 3 (Rong et al., 2013; Shcherbatko et al., 2016; Flinspach et al., 2017; Moyer et al., 2018), and for the substitutions E1A, E1G and D1I in optimized peptides from NaSpTx 1 (Minassian et al., 2013; Revell et al., 2013; Shcherbatko et al., 2016; Rahnama et al., 2017; Zhang et al., 2018b). A decrease in negative charge at the N-terminal also enhances the lipid affinity of NaSpTx 1 toxins such as HwTx-IV (Henriques et al., 2016), suggesting these optimized peptides may have enhanced lipid binding in addition to any enhanced interactions directly with Na_V_ channels. This is relevant when alterations to Na_V_ channel selectivity are sought, since enhanced lipid binding is unlikely to have less ability to influence Na_V_ channel subtype selectivity (Agwa et al., 2018).

Modifications in the C-terminal region that enhance inhibition of Na_V_ channels often involve the introduction of positive charges (Figure 4A and Table 1). This is observed for ICK peptides belonging to NaSpTx 1 and 3, as for the C-terminal amidation of HwTx-IV and CcoTx-1, the introduction of a NHCH_3_ group in ProTx-II and the substitution D32K in CcoTx-1 (Minassian et al., 2013; Revell et al., 2013; Park et al., 2014; Shcherbatko et al., 2016). An increase in hydrophobicity in the C-terminal region also enhanced the Na_V_ inhibition properties of peptides in NaSpTx 1, especially Y33W in HwTx-IV and Y31W in CcoTx-1 (Revell et al., 2013; Shcherbatko et al., 2016; Rahnama et al., 2017). Interestingly, the reduction in hydrophobicity and introduction of negative charges in the C-terminal of the NaSpTx 3 peptide JZTX-V led to a decrease in Na_V_ affinity (analog I28E), but only at Na_V_1.4 (Moyer et al., 2018). A similar reduction in hydrophobicity in the N-terminal region of the NaSpTx 1 analog F5A-GpTx-1 also decreased Na_V_1.4 inhibition (Murray et al., 2015). As our SAR knowledge of NaSpTx 1 and 3 deepens, we anticipate further opportunities to develop more selective and/or potent inhibitors are expected to emerge.

Structural changes in optimized molecules suggest gain of affinity can be associated with regions of the ICK peptide beyond those associated with the local effects of the substitution. For example, structural rearrangements are observed in the HwTx-IV, JZTX-V and HNTX-I optimized analogs (Figure 4B–D) that alter the surface structure of wild-type vs. optimized peptide, with pronounced changes in the N-termini of JZVTX-V with the introduction of Pra (Figure 4C) and following the introduction of W7 in HNTX-I (Figure 4D). However, the three-dimensional structure of a NaSpTx inhibitor bound to a Na_V_ channel embedded in the cell membrane is still to be elucidated.

## The Phylogeny of NaSpTx Targeting Na_V_1.7

**FIGURE 5:**
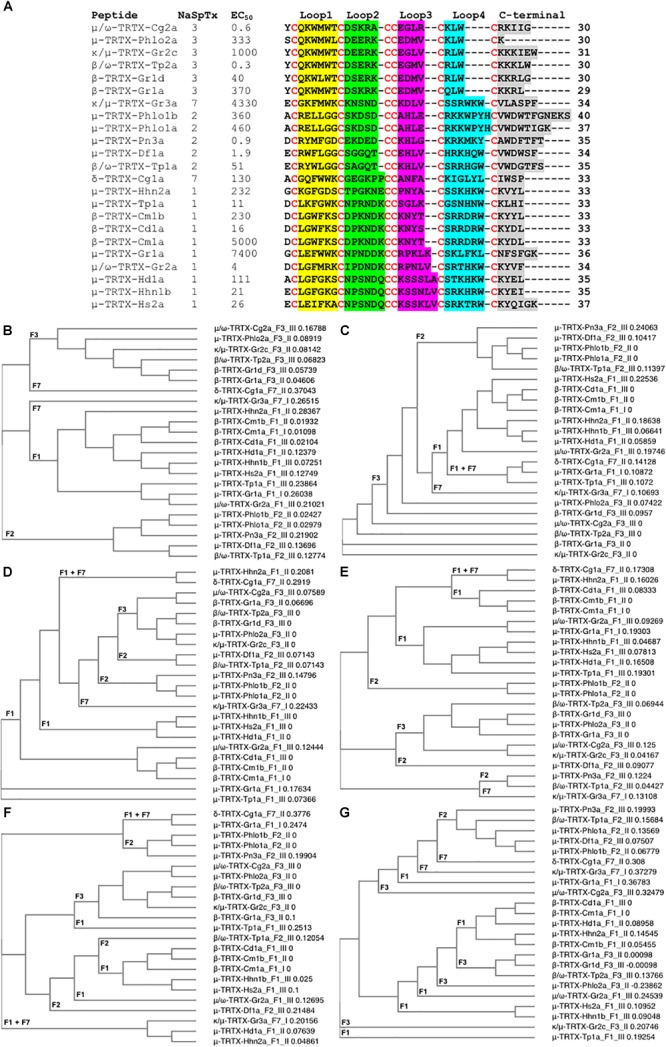
Primary amino acids sequence alignment and phylogenetic analysis of NaSpTx peptides with described Na_V_1.7 modulatory activity. **(A)** Primary sequences alignment of members of NaSpTx1, 2, 3, and 7, with EC_50_ values represented in nM, Loops, 1, 2, 3, 4, and C-terminal shaded in yellow, green, pink, (*blue and gray, respectively, and cysteines colored in red. **(B)** Phylogenetic analysis of the full primary sequences of NaSpTx NaSpTx1, 2, 3, and 7, followed by Loop 1 **(C)**, Loop 2 **(D)**, Loop 3 **(E)**, Loop 4 **(F)**, and C-terminals **(G)**. For the phylogenetic trees, the peptides names are followed by their respective NaSpTx families represented by F1, F2, F3, and F7, and their Na_V_1.7 potency represented by I (EC_50_ > 1 μM), II (EC_50_ between 1 and 0.1 μM), and III (EC_50_ < 0.1 μM). These analyses were performed using Clustal Omega (Sievers et al., 2011) and Simple Phylogeny (Saitou and Nei, 1987). The loops were flanked by cysteine for these analyses.*)

In addition to the SAR studies of the nine NaSpTx discussed above, a number of other NaSpTx display interesting Na_V_1.7 modulatory properties (Figure 5). To date, twenty-three other peptides belonging to NaSpTx 1, 2, 3, and 7 are described with potencies ranging from 0.3 nM to 7.4 μM for half-maximal activity for Na_V_1.7 (Figure 5A). These include μ-TRTX-Pho1a, μ-TRTX-Pho1b and μ-TRTX-Pho2a (Chow et al., 2015), κ/μ-TRTX-Gr2c, κ/μ-TRTX-Gr3a, μ-TRTX-Gr1a, β-TRTX-Gr1d and β-TRTX-Gr1a (Redaelli et al., 2010), μ-TRTX-Pn3a (Deuis et al., 2017), μ-TRTX-Df1a (Cardoso et al., 2017), β/ω-TRTX-Tp2a (Priest et al., 2007), δ-TRTX-Cg1a (Xiao et al., 2005), μ-TRTX-Tp1a (Cardoso et al., 2015), β-TRTX-Cm1b and β-TRTX-Cd1a (Bosmans et al., 2006; Sousa et al., 2017) and μ-TRTX-Hd1a (Klint et al., 2015b). Amongst these, only δ-TRTX-Cg1a activates Na_V_1.7, while the other β- and μ-toxins are Na_V_1.7 inhibitors. Although detailed SAR studies on these toxins have not been described, their sequences similarities make a detailed analysis of their primary structure using phylogenetic approaches useful.

Not surprisingly, NaSpTxs targeting Na_V_1.7 show independent phylogenetic origins and generate well defined clades for NaSpTx 1, 2, and 3, while NaSpTx 7, with only two representatives, clustered as outliers (Figure 5B). These results support previous findings underpinning the classification of the NaSpTx into 12 families (Klint et al., 2012). However, examining relatedness within loops revealed some interesting differences. For example, loop 1 showed similar clustering as the complete primary sequences except NaSpTx 7 clustered with NaSpTx 1 (Figure 5C), while loop 2 had more complex phylogenetic origins, with a primitive ancestor possibly arising from NaSpTx 1 (Figure 5D). Loop 3 also showed complex phylogenetic origins. It was divided into a major clade including the NaspTx1, two members of NaSpTx 2 and one member of NaSpTx 7, a second clade containing NaSpTx 3 and one member of the NaSpTx 2 as outlier, and a small clade including NaSpTx 2 and 7 (Figure 5E). These observations suggest a less purifying selection in loop 3, with NaSpTx 3 remaining the most conservative loop 3 group. The complexity of the phylogenetic origins of the loop 4 and C-terminal regions are considerably higher compared to loops 1, 2, and 3 (Figure 5F,G). Loop 4 diverged into four clades containing a mix of NaSpTx members, and again the NaSpTx 3 clustered further in a single clade, but now with one member of the NaSpTx 1 as an outlier. Finally, the C-terminal was diverged into three major clades that also contained a mix of NaSpTx members. Interestingly, the C-terminals of NaSpTx 2 clustered further into a single clade, indicating that a more conservative evolutionary pressure is occurring in this region of the NaSpTx 2 members.

Overall, we observed that the NaSpTx 1, 2 and 3 families have independent phylogenetic origins. For the NaSpTx 7, more representatives of this family are essential for an appropriated phylogenetic analysis. Remarkably, the loops 2, 3, and 4 forming the peptides in NaSpTx 3 are under a more conservative pressure compared to NaSpTx 1 and 2. This agrees with the SAR studies for ProTx-II (β/ω-TRTX-Tp2a), where optimization was possible only with modifications located in less conserved regions, the C-terminal and in the loop 1, while modifications in loops 2, 3, and 4 were often deleterious for Na_V_ inhibition (Priest et al., 2007; Smith et al., 2007; Schmalhofer et al., 2008; Park et al., 2014; Flinspach et al., 2017). For NaSpTx 1, loop 2 was more conserved and appears to be in agreement with the SAR of CcoTx1 (β-TRTX-Cm1a) where modifications to loop 2 were not well tolerated, while loops 3, 4 and C-terminal tolerated multiple modification (Shcherbatko et al., 2016). Similarly, the C-terminal of NaSpTx 2 was also more conserved compared to the other NaSpTx families, but studies of SAR aiming to unravel the role this C-terminal region in Na_V_ inhibition are yet to be pursued. These patterns of evolutionary pressure provide clues to further explore the SAR of loops and C-terminal residues to that can help further expand the SAR of these Na_V_ channel toxins. However, a simple correlation between phylogenetic origins and potency at Na_V_ channels remains to be established.

## Perspectives in Therapeutic Development

Advances in understanding disease mechanisms and associated novel therapeutic targets position spider ICK peptides as novel drug leads of sufficient size and pharmacophore complexity to restrict their *in vivo* distribution and limit off-target effects. To fully exploit this potential, a deeper understanding of ICK peptide SAR and bio-engineering is required to address specific therapeutic needs. This undoubtedly involves bio-activity and three-dimensional structural determinations for a detailed view of the SAR features. Pre-clinical studies in rodent pain models showing reversal of different types of pain have been reported for spider toxins, including several lacking published SAR data (Table 2).

**Table 2:** Therapeutical potential of NaSpTx peptides evaluated in pre-clinical rodent pain models.

Peptide	NaSpTx family	Spider species	Preferred Na_V_ subtypes	Therapeutic potential	References
Gr1b (GsAFI) Gr2c (GsAFII)	3	*Grammostola rosea*	Na_V_1.7 > 1.4 > 1.1 > 1.2 Na_V_1.7 > 1.4 > 1.1	Acute and inflammatory pain	Lampe, 1998
HwTx-IV	1	*Ornithoctonus huwena*	Na_V_1.7 > 1.2 > 1.3 > 1.4	Inflammatory and SNI-induced neuropathic pain	Liu et al., 2014b
HnTx-IV	1	*Haplopelma hainanum*	Na_V_1.2 > 1.3 > 1.7	SNI-induced neuropathic and formalin-induced inflammatory pain.	Liu et al., 2014a
ProTx-II	3	*Thrixopelma Pruriens*	Na_V_1.7 > 1.6 > 1.2 > 1.5 > 1.3 > 1.8	Painful diabetic neuropathy Inflammatory pain	Tanaka et al., 2015; Flinspach et al., 2017
Hl1a	7	*Haplopelma lividum*	Na_V_1.8	Inflammatory and neuropathic pain	Meng et al., 2016
Pn3a	2	*Pamphobeteus nigricolor*	Na_V_1.7 > 1.1	Inflammatory (with opioid co-administration) and post-surgical pain	Deuis et al., 2017; Mueller et al., 2019
Ca1a	Unknown	*Cyriopagopus albostriatus*	Na_V_1.7 > 1.2 > 1.6 > 1.4 > 1.3	Inflammatory and thermal pain	Zhang et al., 2018c
Ca2a	1	*Cyriopagopus albostriatus*	Na_V_1.7 > 1.2 > 1.6 > 1.3	Inflammatory and thermal pain	Zhang et al., 2018a
Cyriotoxin-1a	1	*Cyriopagopus schioedtei*	Na_V_1.1 > 1.2 > 1.6 > 1.7 > 1.3	Thermal pain	Goncalves et al., 2019

*The NaSpTx peptide with respective family, spider species, preferred voltage-gated sodium channels (Na_*V*_) subtypes and the pre-clinical pain model showing therapeutic efficacy are described. The preferred Na_*V*_ subtypes for some of these NaSpTx peptides were described in: Gr1b and Gr2c (Redaelli et al., 2010), HwTx-IV (Minassian et al., 2013), HnTx-IV (Cai et al., 2015), and ProTx-II (Middleton et al., 2002).*

The NaSpTx3 peptides Gr1b and Gr2c reversed acute and inflammatory pain intrathecally (Lampe, 1998), while ProTx-II and its optimized analog JNJ63955918 reversed neuropathic and inflammatory pain when administered intrathecally or locally (Tanaka et al., 2015; Flinspach et al., 2017). The NaSpTx1 peptides HwTx-IV and HNTX-IV also reversed neuropathic and inflammatory pains intraperitonially (Liu et al., 2014a,b), while ides Ca2a and cyriotoxin-1a reversed inflammatory and thermal pain following intraperitoneal or intraplantar administration, respectively (Zhang et al., 2018a; Goncalves et al., 2019).

In contrast, the NaSpTx2 peptide Pn3a reversed inflammatory pain but only when co-administered with an intraperitoneal opioid (Deuis et al., 2017), despite reversing post-surgical pain following intraperitoneal or local administration (Mueller et al., 2019). Finally, the unclassified spider peptide Ca1a reversed inflammatory and thermal pain following intraperitoneal or intraplantar administrations, respectively (Zhang et al., 2018c). This review of the continuing advancements in the SAR of spider venom ICK peptides will hopefully facilitate efforts to optimize Na_V_ channel modulators for the treatment of complex channelopathies, including different forms of chronic pain.

## Author Contributions

FC performed the phylogenetic analysis, bibliography research, wrote the manuscript, and prepared the figures. RL provided scope, guidance and critically reviewed the manuscript.

## Conflict of Interest Statement

The authors declare that the research was conducted in the absence of any commercial or financial relationships that could be construed as a potential conflict of interest.

## Abbreviations

ASIC: acid sensing channels
Ca_V_: voltage-gated calcium channel
CyA: β-cyanoalanine
DRG: dorsal root ganglion
ICK: inhibitory cysteine knot
K_V_: voltage-gated potassium channel
mAChR: muscarinic acetylcholine receptor
nAchR: nicotinic acetylcholine receptor
Na_V_: voltage-gated sodium channels
Na_V_PaS: insect Na_V_ channel
NET: norepinephrine transporter
Pra: propargylglycine
PyroE: pyroglutamate
SAR: structure-activity relationship
TRP: transient receptor potential channels
TTX-R: tetrodotoxin-resistant
TTX-S: tetrodotoxin-sensitive
VGIC: voltage-gated ion channels
VSD: voltage-sensor domain
